# Assessment of Daily Food and Nutrient Intake in Japanese Type 2 Diabetes Mellitus Patients Using Dietary Reference Intakes

**DOI:** 10.3390/nu5072276

**Published:** 2013-06-26

**Authors:** Yukiko Kobayashi, Mikako Hattori, Sayori Wada, Hiroya Iwase, Mayuko Kadono, Hina Tatsumi, Masashi Kuwahata, Michiaki Fukui, Goji Hasegawa, Naoto Nakamura, Yasuhiro Kido

**Affiliations:** 1 Graduate School of Life and Environmental Sciences, Kyoto Prefectural University, Shimogamo, Sakyo, Kyoto 606-8522, Japan; E-Mails: kbysykk@gmail.com (M.H.); poisson@kpu.ac.jp (S.W.); hgjwj757@yahoo.co.jp (H.T.); kuwahata@kpu.ac.jp (M.K.); kido@kpu.ac.jp (Y.K.); 2 Department of Endocrinology and Metabolism, Graduate School of Medical Science, Kyoto Prefectural University of Medicine, Kajii-cho, Kamigyo, Kyoto 602-8566, Japan; E-Mails: iwase@koto.kpu-m.ac.jp (H.I.); mayucom@koto.kpu-m.ac.jp (M.K.); michiaki@koto.kpu-m.ac.jp (M.F.); goji@koto.kpu-m.ac.jp (G.H.); naoto@koto.kpu-m.ac.jp (N.N.)

**Keywords:** type 2 diabetes mellitus, diet, dietary reference intakes, dietary habits, Japanese

## Abstract

Medical nutrition therapy for the management of diabetes plays an important role in preventing diabetes complications and managing metabolic control. However, little is known about actual eating habits of individuals with type 2 diabetic mellitus (T2DM), especially in Japan. Therefore, we sought to (1) assess the dietary intake of individuals with T2DM, and (2) characterize their intake relative to national recommendations. This cross-sectional study involved 149 patients (77 males and 72 females) aged 40–79 years with T2DM recruited at a Kyoto hospital. Dietary intake was assessed using a validated self-administered diet history questionnaire. Under-consumption, adequacy, and over-consumption, of nutrients were compared to the age- and sex-based standards of the Japanese Dietary Reference Intakes. Among the results, most notable are (1) the inadequacy of diets in men with respect to intake of vitamins and minerals, likely owing to low intake of vegetables and fruits; (2) excess contributions of fat intake to total energy in both sexes; and (3) excess consumption of sweets and beverages relative to the national average. The prevalence of diabetes complications may be increasing because of a major gap between the typical dietary intake of individuals with T2DM and dietary recommendation.

## 1. Introduction

The prevalence of type 2 diabetic mellitus (T2DM) has been increasing throughout the world [1], including Japan. The number of Japanese patients with diabetes or in a pre-diabetes state (HbA1c ≥ 5.5%) is estimated to be about 22.1 million, with an increase of about 5.9 million in the 5-year period ending in 2007 [2]. Medical nutrition therapy for the management of diabetes plays an important role in preventing complications associated with diabetes, especially the management of metabolic control and optimal weight [3,4]. The European Diabetes Association stated that carbohydrate intake should make up 45%–60% of the total energy, and that while there is no justification in advising an extreme carbohydrate-restricted diet, lipid intake should not exceed 35% [5,6]. In recommendations published in 2012, the American Diabetes Association reported that glycemic control can be improved by controlling carbohydrate intake, and that either low-carbohydrate, low-fat calorie-restricted, or Mediterranean diets may be effective for weight loss if followed for up to two years [7]. The guidelines for the treatment of diabetes in Japan recommend a diet based on the following: carbohydrates comprising 50%–60% of the total energy intake, protein 1.0–1.2 g/kg of the ideal body weight, and fat ≤25% of the total energy intake [8].

The nutritional recommendations for treatment of diabetes in Japan are disseminated mainly by medical professionals. For example, one of the nutritional education tools for T2DM patients that has been widely employed is guidance based on the “Food Exchange Lists” edited by the Japan Diabetes Society [9]. The Food Exchange Lists consist of six different lists. In each of these lists, 1 unit is the equivalent of 80 kcal, because for many commonly eaten foods, a single serving is equivalent to about 80 kcal or a multiple thereof. The lists have been designed so that food servings within the same list can be exchanged with each other, as long as the number of units is not changed. A food classification table gives the weighted average amount of nutrients for each list. The first edition of the Food Exchange Lists was published in 1965 and has since been repeatedly revised. The newest lists focus on control of energy intake [10].

However, there are many cases in clinical practice where dietary selections and habits of T2DM patients are not in line with recommendations, despite the fact that the utility of nutritional therapy for diabetes has been firmly established. We hypothesize that the observed increasing prevalence of complications associated with diabetes [11] has been increasing because of the major gap between the usual dietary habits of T2DM patients and dietary therapy recommendations. However, little is known about the actual eating habits of Japanese with T2DM. In this study, we sought to assess the dietary intake of individuals with T2DM, and characterize their intake relative to the Dietary Reference Intakes (DRIs) for Japanese and the average Japanese dietary intake.

## 2. Methods

### 2.1. Study Participants

We enrolled T2DM patients treated in the University Hospital, Kyoto Prefectural University of Medicine in the study. A self-administered diet history questionnaire (DHQ) was given to 260 patients (140 males and 120 females). A total of 215 patients (112 males and 103 females) completed the questionnaire, yielding a collection rate of 82.7%. A total of 149 patients (77 males and 72 females) met the inclusion criteria, after exclusions due to the following criteria: under the age of 40 (4 females), type 1 diabetes mellitus patients (7 males and 6 females), chronic renal failure or hemodialysis patients (7 males and 4 females), incomplete information (12 males and 6 females), and participants in the ≥95 or ≤5 percentile for energy intake (10 males and 10 females). The data were collected from June to September 2011. Each participant agreed to participate in the study by signing an informed consent form before entering the study. The experimental protocol was approved by the ethics committee of Kyoto Prefectural University of Medicine (Kyoto, Japan).

### 2.2. Estimation and Assessment of Habitual Food and Nutrient Intake

The usual dietary habits of the participants during the one-month period preceding the study were assessed with the validated DHQ system [12]. The questionnaire employed in this system consists of questions regarding general dietary behavior, major cooking methods, the quantity and frequency of consuming 149 selected food and beverage items, and the amount of rice and miso soup consumed daily. The food and beverage items and portion sizes in the questionnaire were derived primarily from the data in the National Nutrition Survey of Japan (NNSJ) [13] and several recipe books for Japanese dishes. We determined the intake of food groups (grains and cereals, potatoes, sugar, pulses, nuts, vegetables, green vegetables, white vegetables, fruits, mushrooms, algae, fish and shellfish, meat, eggs, milk, oils and fats, confectionery, and beverages), and nutrients (protein, animal protein, fat, animal fat, cholesterol, carbohydrates, fiber, vitamin A, β-carotene, vitamin D, E, K, B1, B2, niacin, vitamin B6, B12, folic acid, pantothenic acid, vitamin C, salt, potassium, calcium, magnesium, phosphorus, iron, zinc, and copper), total energy, total and percentage of energy from protein, carbohydrates, and fat, and the animal protein ratio per day using a dedicated program for the DHQ system (DHQ BOX system 2008, Gender Medical Research, Tokyo, Japan).

Assessment of the adequacy of the estimated energy and nutrient intake was determined using the estimated average requirement (EAR), the adequate intake (AI), or the tentative dietary goal for preventing lifestyle-related diseases (DG) within the DRIs for Japanese [14]. The following 14 items were assessed using the EAR: protein, vitamin A, B1, B2, niacin, vitamin B6, B12, folic acid, vitamin C, calcium, magnesium, iron, zinc and copper. The following five items were assessed using the AI: vitamin D, E, K, pantothenic acid, and phosphorus. The following five items were assessed using the DG: fat and carbohydrates energy ratio, fiber, salt, and potassium. The percentages for patients who showed intakes less than the EAR or AI to the whole were presented for individual nutrients. The percentages for patients who showed intakes less and greater than the DG to the whole were presented for individual nutrients. For these calculations, the EAR and AI values and DG ranges for the age classes (40–49, 50–69, and over 70 years) of each patient were used. The average values for nutrient and food group intakes were compared with the results for those aged 60–69 years old in the NNSJ, using the participants’ average age. The NNSJ has been conducted since 1946, and is the only nationwide data source of nutritional aspects in Japanese adults [15]. By comparing with the NNSJ, we investigated whether there would be any difference between the intake of the participants and the average Japanese.

### 2.3. Participants’ Physical Status, Medication, and Blood Constituent Values

Data on the physical status, medication, and blood constituent values of the participants were taken from the electronic medical records. Regarding the physical status and medication, we obtained the following data: age, height, weight, body mass index (BMI), and the administration method of the treatment employed (oral diabetes medication, dyslipidemia medication, and/or insulin). The blood constituent values of the participants were obtained for the following 7 items: HbA1c (*n =* 149), plasma glucose (*n =* 149), triglycerides (*n =* 145), LDL-cholesterol (*n =* 99), HDL-cholesterol (*n =* 146), urea nitrogen (*n =* 146), and creatinine (*n =* 146).

### 2.4. Statistical Analysis

The relationships between the males and females were evaluated by age classes and the administration of diabetes and dyslipidemia medication and/or insulin using the χ^2^-test, as well as BMI, HbA1c, plasma glucose, triglycerides, LDL and HDL-cholesterol, urea nitrogen, and creatinine using the Mann-Whitney U test. We calculated the differences between the NNSJ and the values obtained from the participants, and assessed them using the 95% confidence interval and *p*-values. All analyses were performed with PASW statistics version 18.0 (IBM, Chicago, IL, USA). The level of significance was set at *p* < 0.05.

## 3. Results

### 3.1. Patient Characteristics

Significant differences were demonstrated between the males and females in the administration of oral dyslipidemia medication, and the serum triglycerides, LDL and HDL–cholesterol, and creatinine values. No significant differences were demonstrated between the age classes, the administration of diabetes medicine or insulin, or the HbA1c, serum glucose, and urea nitrogen values. Over half of the patient samples were shown above the control target for the prevention of diabetes-related complications of HbA1c (<7.0%, according to The Japan Diabetes Society [8]) for both the males and females (Table 1).

**Table 1:** Characteristics of type 2 diabetic mellitus (T2DM) patients.

		Male	Female	*p* value
		*n* = 77	*n* *=* 72	
Age (years)		65.3 ± 9.2	66.1 ± 9.5	0.74
Age classes, *n* (%)	40–49	6 (7.8)	6 (8.3)	1.00
	50–59	11 (14.3)	10 (13.9)	
	60–69	32 (41.6)	29 (40.3)	
	over 70	28 (36.4)	27 (37.5)	
Diabetes medicine, yes, *n* (%)		59 (77)	60 (83)	0.41
Dyslipidemia medicine, yes, *n* (%)		26 (34)	38 (53)	0.02
Insulin treatment, yes, *n* (%)		13 (17)	20 (28)	0.12
BMI (kg/m^2^)		24.3 ± 3.9	23.7 ± 4.5	0.23
HbA1c (%)		6.8 ± 0.9	6.9 ± 0.9	0.34
(≥7.0 *, %)		(58.4)	(58.3)	
Plasma glucose (mg/dL)		157 ± 50	152 ± 59	0.40
(≥180 *, %)		(28.6)	(25.0)	
Triglycerides (mg/dL)		149 ± 92	120 ± 77	0.03
(≥150 *, %)		(37.3)	(21.4)	
LDL cholesterol (mg/dL)		114 ± 22	98 ± 22	0.01
(≥120 *, %)		(45.5)	(15.4)	
HDL cholesterol (mg/dL)		55 ± 14	64 ± 16	<0.01
(<40 *, %)		(10.7)	(1.4)	
Urea nitrogen (mg/dL)		15.8 ± 4.2	15.4 ± 4.4	0.31
Creatinine (mg/dL)		0.84 ± 0.19	0.62 ± 0.23	<0.01

Mean ± SD. Relationships between males and females were evaluated by age classes and administration of diabetes, dyslipidemia medicine and insulin using the χ^2^-test, and BMI, HbA1c, serum glucose, triglycerides, LDL and HDL-cholesterol, urea nitrogen, and creatinine using the Mann-Whitney U test; * Control targets for the prevention of diabetes-related complications according to The Japan Diabetes Society [8].

### 3.2. Assessment of Nutrient Intake Using the DRIs

**Figure 1:**
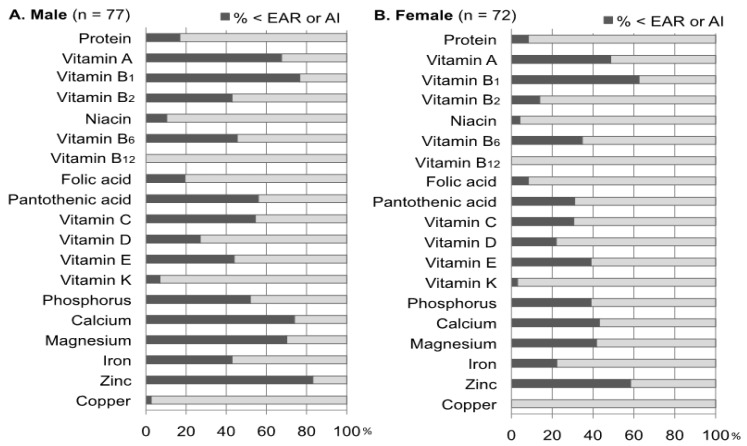
Assessment of the nutrient intake using the estimated average requirement (EAR) and adequate intake (AI) within Dietary Reference Intakes (DRIs) for Japanese. The percentages of patients who showed intakes less than the EAR or AI to the whole were presented for individual nutrients. For these calculations, the EAR or AI values for the age classes (40–49, 50–69, and over 70 years) of each patient were used.

Over half of T2DM patients reported consuming less than the EAR of the following nutrients: vitamin A (% < EAR: 68%), B1 (77%), C (55%), calcium (74%), magnesium (70%), and zinc (83%) in males, as well as vitamin B1 (63%) and zinc (58%) in females. In addition, over half of male T2DM patients also reported consuming less than the AI for pantothenic acid (56%) and phosphorous (52%). On the other hand, the results showed that there were almost no participants with vitamin B12 or copper inadequacy in either both the male and female groups (Figure 1).

**Figure 2:**
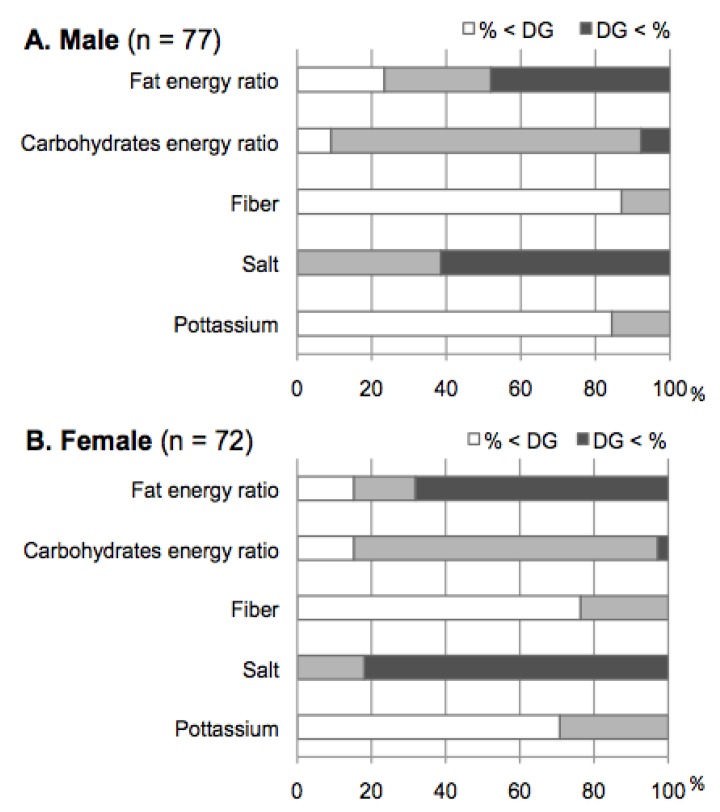
Assessment of the nutrient intake using the tentative dietary goal for preventing life-style related diseases (DG) within the Dietary Reference Intakes (DRIs) for Japanese. The percentages for patients who showed intakes less and greater than the DG to the whole were presented for individual nutrients. For these calculations, the DG ranges for the age classes (40–49, 50–69, and over 70 years) of each patient were used.

More than 80% of male and female T2DM patients were meeting DG targets for carbohydrates as a percentage of energy. In regard to the percentage of energy from obtained from fat, the percentage of those who were over the DG range were 48.1% in the male group and 68.1% in the female group, respectively. On the other hand, a majority of both the males and females did not reach the DG recommendations for fiber and potassium, and exceeded the DG for salt (Figure 2).

### 3.3. Comparisons between Food Group or Nutrients Intake and the NNSJ

In the male participants, the food groups showing intakes significantly higher than the NNSJ, that is, higher than the average Japanese intake, were milk, oils and fats, confectionery, and beverages, while the items that were significantly lower, that is, less than the average Japanese intake, were grains and cereals, potatoes, pulses, vegetables, fruit, fish and shellfish, and meat. There were no nutrients that were significantly higher, compared with the NNSJ, while the items that significantly lower were energy, protein, carbohydrates, potassium, calcium, magnesium and phosphorus (Table 2). In the female group, the food groups that were significantly higher were green vegetables, milk, oils and fats, confectionery, and beverages, while the items that were significantly lower were grains, cereals and potatoes. There were no nutrients that were significantly higher, compared with the NNSJ, but one item was significantly lower, the carbohydrates energy ratio (Table 3). There were many items showing low values in the male group, compared with the female group.

**Table 2:** The intake of food groups and nutrients in male patients, and comparison with the National Nutrition Survey of Japan (NNSJ) (per day).

Male	Patients (*n* = 77)	NNSJ (*n* = 726)	Difference of NNSJ from Patients			
	Mean	Mean	Mean	95%CI		*p* value
Grains and Cereals (g)	437	513	−76	−114.8	−37.5	<0.01
Potatoes (g)	27	57	−30	−48.3	−11.2	<0.01
Sugar (g)	13	8	5	−1.8	12.7	0.14
Pulses (g)	4.5	73	−28	−50.0	−6.9	0.01
Nuts (g)	2.6	3.0	−0.4	−13.0	12.3	0.96
Vegetables (g)	266	318	−52	−100.7	−3.2	0.04
Green vegetables (g)	115	105	11	−16.4	38.0	0.44
Write vegetables (g)	150	187	−37	−70.2	−3.5	0.03
Fruits (g)	94	126	−32	−60.9	−3.1	0.03
Mushrooms (g)	11	22	−10	−25.0	4.2	0.16
Algae (g)	10	13	−3	−15.5	9.0	0.61
Fishes and shellfishes (g)	85	107	−23	−42.0	−3.2	0.02
Meats (g)	55	77	−22	−38.7	−5.2	0.01
Eggs (g)	38	37	1	−12.2	13.3	0.94
Milk (g)	137	91	46	6.7	84.6	0.02
Oils and fats (g)	19	10	9	2.8	15.0	<0.01
Confectionery (g)	46	20	27	7.6	45.5	0.01
Beverages (g)	1029	828	201	71.1	330.6	<0.01
Energy (kcal)	1925	2143	−218	−302	−134	<0.01
Protein (g)	68	78	−10	−16.3	−3.2	<0.01
Animal protein (%)	52	51	2	−2.8	5.9	0.48
Fat (g)	53	55	−2	−8.7	5.3	0.64
Fat (E%)	25	23	2	−1.1	4.8	0.22
Animal fat (g)	24	27	−3	−9.2	3.0	0.31
Cholesterol (mg)	323	334	−11	−46.9	25.3	0.56
Carbohydrates (g)	260	295	−35	−51.6	−18.0	<0.01
Carbohydrates (E%)	54	62	−8	−10.8	−5.5	<0.01
Fiber (g)	13	17	−4	−7.2	0.0	0.05
Salt (g)	10.8	12.0	−1.2	−3.9	1.4	0.36
Potassium (mg)	2343	2591	−248	−416	−80	<0.01
Calcium (mg)	501	561	−60	−112	−7	0.03
Magnesium (mg)	254	285	−31	−51.3	−10.9	<0.01
Phosphorus (mg)	1025	1104	−79	−145	−13	0.02
Iron (mg)	6.9	8.9	−2.0	−4.2	0.1	0.07
Zinc (mg)	7.6	8.9	−1.3	−3.2	0.6	0.17
Copper (mg)	1.07	1.32	−0.25	−0.97	0.47	0.49

Because the associations for vitamins were not significant, the results for vitamins are not shown. 95% CI: 95% Confidence Interval.

**Table 3:** The intake of food groups and nutrients in female patients, and comparison with the National Nutrition Survey of Japan (NNSJ) (per day).

Male	Patients (*n* = 72)	NNSJ (*n* = 815)	Difference of NNSJ from Patients			
	Mean	Mean	Mean	95%CI		*p* value
Grains and Cereals (g)	354	385	−31	−56.9	−5.2	0.02
Potatoes (g)	28	58	−29	−58	−11.0	<0.01
Sugar (g)	13	8	5	−1.7	12.6	0.14
Pulses (g)	62	63	−1	−22.0	19.6	0.91
Nuts (g)	2.6	2.4	0.2	−9.0	9.5	0.96
Vegetables (g)	335	320	15	−49.7	80.0	0.65
Green vegetables (g)	169	111	58	8.3	106.9	0.02
Write vegetables (g)	166	185	−18	−50.0	13.1	0.25
Fruits (g)	128	155	−27	−60.7	7.6	0.13
Mushrooms (g)	13	20	−7	−20.2	5.6	0.27
Algae (g)	10	13	−3	−16.2	10.4	0.67
Fishes and shellfishes (g)	81	86	−4	−23.4	14.8	0.66
Meats (g)	51	61	−10	−26.9	6.8	0.24
Eggs (g)	39	33	6	−12.5	25.0	0.51
Milk (g)	144	110	35	3.4	65.8	0.03
Oils and fats (g)	18	9	9	3.0	15.9	<0.01
Confectionery (g)	67	25	41	19.2	63.4	<0.01
Beverages (g)	1110	654	456	302.6	608.8	<0.01
Energy (kcal)	1171	1732	−21	−119	7.6	0.67
Protein (g)	66	67	−1	−7.4	5.6	0.78
Animal protein (%)	52	50	2	−2.0	6.6	0.30
Fat (g)	53	49	4	−3.4	12.0	0.28
Fat (E%)	27	25	2	−0.8	5.4	0.14
Animal fat (g)	22	24	−1	−7.3	4.9	0.69
Cholesterol (mg)	293	291	2	−34.2	37.6	0.92
Carbohydrates (g)	237	249	−12	−27.7	3.4	0.13
Carbohydrates (E%)	56	60	−4	−6.3	−0.8	0.01
Fiber (g)	14	17	−2	−6.1	1.2	0.19
Salt (g)	10.3	10.6	−0.3	−3.1	2.4	0.81
Potassium (mg)	2571	2510	61	−163	285	0.59
Calcium (mg)	569	555	14	−38	65	0.60
Magnesium (mg)	261	260	1	−22	23	0.95
Phosphorus (mg)	1022	975	47	−25	118	0.20
Iron (mg)	7.3	8.3	−1.0	−3.3	113	0.41
Zinc (mg)	7.3	7.5	−0.2	−2.1	1.6	0.81
Copper (mg)	1.09	1.15	−0.06	−0.74	0.62	0.86

Because the associations for vitamins were not significant, the results for vitamins are not shown. 95% CI: 95% Confidence Interval.

## 4. Discussion

This study is the first to examine self-reported habitual food and nutrient intake in a group of Japanese T2DM patients relative to the DRIs and to average Japanese dietary intake. Although a statement regarding dietary therapy for Japanese T2DM patients has been published [8], it has not been reflected in the DRIs for T2DM because the evidence for the statement was insufficient. Therefore, we used the DRIs for Japanese, which has been formulated with the goal of preventing lifestyle-related diseases, including T2DM.

Many of the patient samples in the present study showed results suggesting that glycemic control was insufficient or poor without diabetes-related complications, such as dialysis and chronic renal failure. We assessed each participant’s dietary intake by calculating the percentage of the participants below EAR and AI, and those who were outside the DG range in the DRIs. More than half of the participants were below the EAR and AI for seven nutrients in the male group and two nutrients in the female group, demonstrating that the dietary inadequacy for vitamins and minerals in males was greater than that in females. These characteristics appeared to be due to a low intake of vegetables and fruit. Indeed, compared with the NNSJ, the total intake of vegetables and fruit in the male group was significantly lower, and the intake of white vegetables was lower in the female group. Moreover, the majority of the participants had not reached the DG for fiber and potassium, which suggested an inadequate intake of vegetables and fruit. The results of cohort studies showed that a sufficient intake of vegetables and fruit reduced the frequency of developing diabetes [16,17], and a diet high in dietary fiber containing vegetables might improve glycemic control [18,19]. Several studies conducted with elderly Japanese participants with T2DM demonstrated that diets rich in vegetables were correlated with improved HbA1c, serum triglyceride levels [20], and life prognosis [21]. Therefore, it is important to educate Japanese T2DM patients about the health advantages of consuming vegetables.

The present study suggested that many T2DM patients had a high-fat energy ratio. One contributing factor was the intake of fats and oils as well as milk in both males and females being significantly higher, compared with the NNSJ. On the other hand, the median carbohydrate energy ratio was 55–56 E% in our participants, which was comparatively low, although it was within the range of the DG (50 < E% < 70). A relatively low-carbohydrate energy ratio in patients seemed to be caused by a low intake of grains and cereals, and potatoes, compared with the NNSJ. Therefore, many of the patients in the present analysis may practice modest carbohydrate dietary patterns as part of their dietary therapy, resulting in a high-fat energy ratio. Several studies in Japan have demonstrated that a dietary pattern with a frequent intake of white rice was related to the deterioration of the glycemic metabolism [22,23], and associated with an increased risk of T2DM [24,25]. Currently, discussions are in progress regarding an appropriate carbohydrate intake, since diabetes improvement with a low-carbohydrate diet has shown results [26]. Alternatively, it has been demonstrated that a high-fat/low-carbohydrate diet increased the LDL-cholesterol level, although there was body weight loss, an increase in HDL-cholesterol, and an decrease in triglyceride levels compared with a low-fat/high-carbohydrate diet in obese participants with insulin resistance [27,28]. Consequently, no consensus has been reached regarding the optimal fat energy ratio for T2DM patients. Therefore, future studies should be conducted to investigate whether or not energy from the intake of fats is compensated by a low intake of carbohydrates.

On the other hand, the intake of confectionery, sugars, and beverages in our participants was higher than the NNSJ, despite the fact that they had been instructed to eat fewer sweets containing high levels of sugar. A dietary pattern with a high consumption of beverages and confectionery reportedly can lead to increased insulin resistance and the development of diabetes [29,30]. As the patients in the present analysis self-reported a strong preference for confectionary and beverages, it may be necessary to more carefully monitor intake of these foods for adherence to recommendations.

We clarified the dietary intake of Japanese individuals with T2DM and studied the features of their dietary intake. However, there were several limitations to the present study. We speculated that there may have been selection bias, as these patients self-selected into the study and are therefore likely interested in their diet. Further, as this study was conducted within the context of a hospital where the patients receive treatment, they may have under-reported intake of less desirable foods and over-reported intake of healthier foods in order to satisfy researchers. While significant correlations were demonstrated for many items in the male group, there were only a few items that showed significant correlations in the female group, which might have been due to erroneous declarations. In the present study, the mean values of nationally estimated intake were cited from the latest version of the annually published NNSJ. The SEs were calculated from the SDs. Ideally, future studies will generate estimates of national intake directly using NNSJ data, as opposed to published estimates, when/if that data becomes available. Further, at that time, estimates of dietary adequacy in the population as a whole may also be used for comparison with the estimates of dietary in/adequacy reported here for T2DM patients. In addition, we did not assess the alcohol intake of the participants in this study. It is known that alcohol affects diabetes incidence but in a manner that is different from those of other beverages [31]. In future studies, alcohol intake should also be considered in order to correctly assess the diet-disease relationships.

## 5. Conclusions

We assessed the dietary intake of Japanese T2DM patients, and identified the following three features. First, there was a low intake of vitamins and minerals in the male participants, which was probably related to the low intake of vegetables and fruits. Second, there was a high fat energy ratio due to not only a modest carbohydrate diet, but also a high intake of fats and oils. The third feature was a higher consumption of confectionery and beverages, which the subjects had been instructed to consume in moderation. Therefore, the prevalence of complications associated with diabetes may be increasing because of a major gap between the usual dietary habits of patients and dietary therapy. It can be considered that these features will be incorporated in the dietary therapy data pool and nutritional education, and therapy guidance for Japanese T2DM patients should reflect these results in the future.
